# New York Academy of Medicine

**Published:** 1894-06

**Authors:** D. B. St. John Roosa


### New York Academy of Medicine.

D. B. ST. JOHN ROOSA, M.D., PRESIDENT.

### Scurvy in Children.

Dr. W. P. Northrup read a paper with this title. He said: The first case of scurvy I ever saw died, but the autopsy in this case did something to advance our knowledge of this subject. In 1873, a case of infantile scurvy in a child eighteen months old was reported by a continental observer. In the *Lancet* for November, 1878, by W. B. Cheever, of London, appeared three cases of scurvy supervening upon rachitis in young children. The writer first saw a case of this kind in the New York Foundling Asylum in 1889. It was not known to have scurvy, and did not receive proper dietetic treatment. The least affected leg in this case showed hemorrhage about the femur and tibia. The other leg was very much worse, the lower end of the shaft of the femur was separated from the epiphysis, and the bone looked as if it had been boiled for some time. This case was reported to the New York Pathological Society in 1889. The diagnosis was made from the post-mortem findings. Two years later the writer met with a typical case in consultation. The patient had the best of surroundings, and the case was diagnosticated early and promptly treated, so that it was well in thirty days. I then collected in New York City and elsewhere nine other cases.

Scurvy is plainly a disease of mal-nutrition dependent upon long use of improper food, and the absence of fresh food or fresh fruit juice. Its most characteristic symptoms are spongy gums and a tendency to hemorrhages—notably sub-periosteal collections of blood about the femur and other long bones. There may be hematemesis, hematuria, and bloody stools.

A child sixteen months old, surrounded by everything wealth could bring; both parents extremely robust and healthy. The child was breast fed for a few months, and was then fed on "prepared babies' food." Dentition occurred at the usual time, but the child was backward in walking. After being fed for twelve months on this food, the gums were noticed to be unhealthy looking, and a week later the joints of one extremity became swollen and tender. When seen in consultation, the gums were very much swollen and hemorrhagic. It could move the right leg only slightly, and the right thigh was the seat of a fusiform swelling just above the knee. There were no other external manifestations. The child was sent to the country and given fresh cows' milk, and orange-juice. The latter was taken greedily. Improvement was immediate, so that in ten days the gums were entirely normal, and the thigh was very much better. One month later the child was standing on its feet.

In my paper on this subject, read at Washington, in September, 1891, I reported twelve cases of scurvy in children. In eight of the cases the age of the children ranged from eleven to eighteen months; the others were from two and a half to six years of age. The temperature in these cases did not exceed 101°. Various causes were assigned—prepared food, condensed milk, exclusive feeding for some time on prepared food, and exclusive diet of meat for three months. One observer states that in two cases he succeeded in isolating a slightly curved bacillus; and when rabbits were inoculated with these bacilli there were extravasations of blood in various parts of the body, but the organism could not be found in the fluids of the body. Inoculations with pure cultures of these bacilli in large quantity, gave rise to abscesses and death. The reporter expresses the opinion that the organism is ordinarily found in the mouth, and in cases of mal-nutrition finds a favorable soil. In the treatment of successful cases orange-juice and fresh milk seem to bring about the most prompt improvement. In one case in which the administration of acids was combined with that of iron, recovery took place, but more gradually. The internal administration of chlorate of potash in one case for several days caused no improvement, but the patient rapidly recovered when given orange-juice which was taken with avidity.

Scurvy is a condition of mal-nutrition which affects the capillaries, allowing the blood to escape from them, probably by diapedesis. This also causes sponginess of the gums. The collections of blood occur most often about the shafts of the femora and beneath the periosteum. The next most frequent are petechiæ and ecchymoses of the skin, often in the eyelids.

The second year of life is the most common period of scurvy. The disease is believed to be a distinct entity, although often associated with or superadded to rickets. Formerly it was believed to be so closely associated with rickets as to justify the name "acute rickets."

